# Supplementary material for: Serial Cultivation of an MSC-Like Cell Line with Enzyme-Free Passaging Using a Microporous Titanium Scaffold
Source: Materials (Basel). 2023 Jan 30;16(3):1165. doi: 10.3390/ma16031165 (PMC9919603; doi:10.3390/ma16031165)
Supplement: Supplementary file 1 [file materials-16-01165-s001.zip › materials-2077294-supplementary.pdf]

materials

Supplementary Materials

Serial Cultivation of an MSC-Like Cell Line with Enzyme-Free Passaging using a Microporous Titanium Scaffold

Yukihiko Sakisaka [1], Hiroshi Ishihata [1,*], Kentaro Maruyama [1], Eiji Nemoto [1], Shigeki Chiba [2], Masaru Nagamine [2], Hiroshi Hasegawa [3], Takeshi Hatsuzawa [4] and Satoru Yamada [1]

[1] Department of Periodontology and Endodontology, Tohoku University Graduate School of Dentistry, 4-1 Seiryou-machi, Aoba-ku, Sendai 980-8575, Japan
[2] Nagamine Manufacturing Co., Ltd., 1725-26, Kishinoue, Manno-cho, Nakatado-gun, Kagawa 766-0026, Japan
[3] Department of Oral Surgery and Dentistry, Fukushima Medical University, 1, Hikariga-oka, Fukushima 960-1295, Japan
[4] Laboratory for Future Interdisciplinary Research of Science and Technology, Institute of Innovative Research, Tokyo Institute of Technology, 4259 Nagatsuta-cho, Midori-ku, Yokohama, Kanagawa 226-8503, Japan
* Correspondence: hiroshi.ishihata.a8@tohoku.ac.jp; Tel.: +81-22-717-8336 or +81-22-717-8339

Citation: Sakisaka, Y.; Ishihata, H.; Maruyama, K.; Nemoto, E.; Chiba, S.; Nagamine, M.; Hasegawa, H.; Hatsuzawa, T.; Yamada, S. Serial Cultivation of an MSC-Like Cell Line with Enzyme-Free Passaging using a Microporous Titanium Scaffold. *Materials* **2023**, *16*, 1165. https://doi.org/10.3390/ma16031165

Academic Editors: Qingquan Liu and Shaohui Xiong

Received: 20 November 2022
Revised: 20 January 2023
Accepted: 26 January 2023
Published: 30 January 2023

Copyright: © 2023 by the authors. Licensee MDPI, Basel, Switzerland. This article is an open access article distributed under the terms and conditions of the Creative Commons Attribution (CC BY) license (https://creativecommons.org/licenses/by/4.0/).

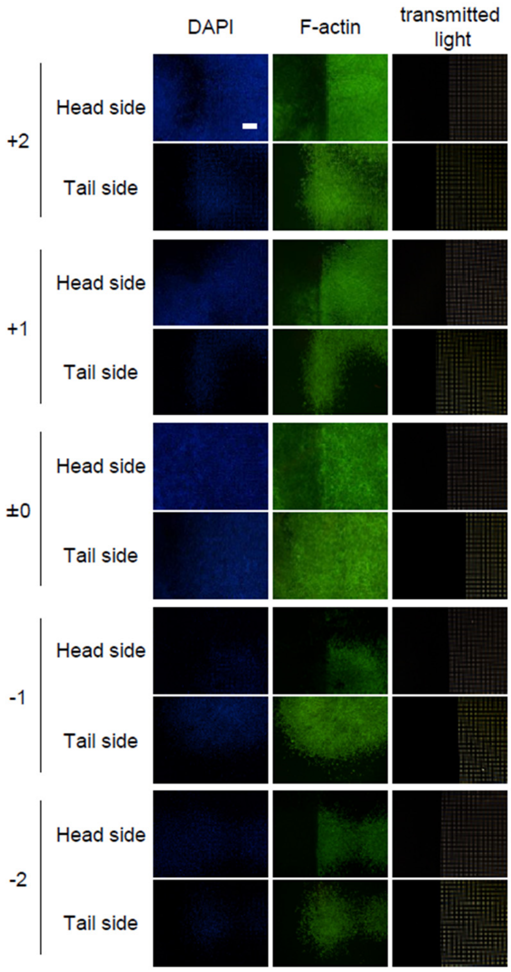

Figure S1. The distributions of C3H10T1/2 cells on Ti membranes. The cells were stained for F-actin (green) and nuclei (blue). Compared with the tail side, F-actin expression was lower outside of the microperforated area than inside of it on the head side (the scale bars represent 750 μm).
